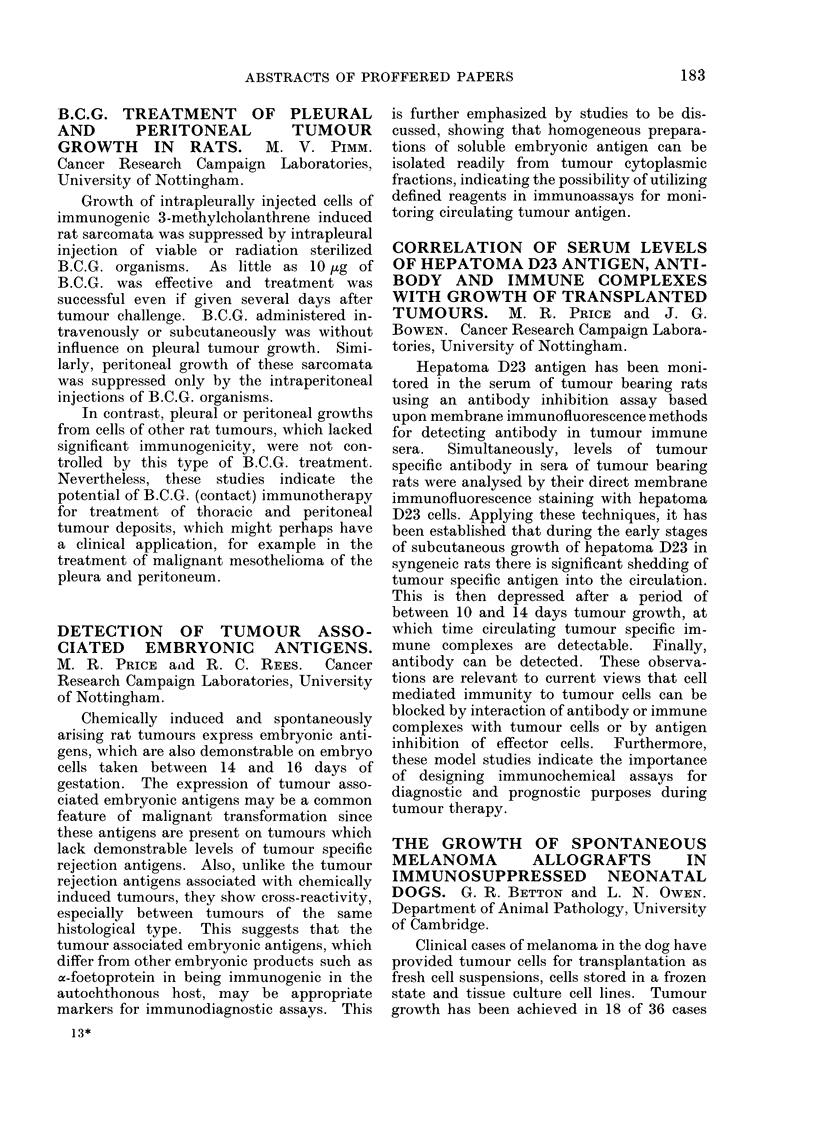# Proceedings: Detection of tumour associated embryonic antigens.

**DOI:** 10.1038/bjc.1974.166

**Published:** 1974-08

**Authors:** M. R. Price, R. C. Rees


**DETECTION OF TUMOUR ASSOCIATED EMBRYONIC ANTIGENS.** M. R. Price and R. C. Rees. Cancer Research Campaign Laboratories, University of Nottingham.

Chemically induced and spontaneously arising rat tumours express embryonic antigens, which are also demonstrable on embryo cells taken between 14 and 16 days of gestation. The expression of tumour associated embryonic antigens may be a common feature of malignant transformation since these antigens are present on tumours which lack demonstrable levels of tumour specific rejection antigens. Also, unlike the tumour rejection antigens associated with chemically induced tumours, they show cross-reactivity, especially between tumours of the same histological type. This suggests that the tumour associated embryonic antigens, which differ from other embryonic products such as α-foetoprotein in being immunogenic in the autochthonous host, may be appropriate markers for immunodiagnostic assays. This is further emphasized by studies to be discussed, showing that homogeneous preparations of soluble embryonic antigen can be isolated readily from tumour cytoplasmic fractions, indicating the possibility of utilizing defined reagents in immunoassays for monitoring circulating tumour antigen.